# Animal model of assessing cerebrovascular functional reserve by imaging photoplethysmography

**DOI:** 10.1038/s41598-020-75824-w

**Published:** 2020-11-04

**Authors:** Oleg V. Mamontov, Alexey Y. Sokolov, Maxim A. Volynsky, Anastasija V. Osipchuk, Valery V. Zaytsev, Roman V. Romashko, Alexei A. Kamshilin

**Affiliations:** 1 Department of Circulation Physiology, Almazov National Medical Research Centre, Saint Petersburg, Russia; 2 Department of Departmental Therapy, Pavlov First Saint Petersburg State Medical University, Saint Petersburg, Russia; 3 Department of Neuropharmacology, Valdman Institute of Pharmacology, Pavlov First Saint Petersburg State Medical University, Saint Petersburg, Russia; 4 Pavlov Institute of Physiology of the Russian Academy of Sciences, Saint Petersburg, Russia; 5 Faculty of Applied Optics, ITMO University, Saint Petersburg, Russia; 6 Laboratory of High-Precision Optical Measurements, Institute of Automation and Control Processes FEB RAS, Vladivostok, Russia; 7 Faculty of Photonics and Optical Information, ITMO University, Saint Petersburg, Russia

**Keywords:** Biological techniques, Biomarkers, Cardiology, Medical research, Optics and photonics

## Abstract

Assessment of the cerebral blood-flow-reserve in patients with cerebrovascular diseases is extremely important in terms of making prognosis, determining treatment tactics, and controlling the revascularization outcome in the case of reconstructive interventions on the brain vessels. However, there is no easy-to-use, contactless method for either assessing the functional reserve of the cortical vascular network or intraoperative monitoring of surgical intervention. Our study aims to demonstrate feasibility of green-light imaging photoplethysmography (iPPG) to estimate cerebrovascular functional reserve in animal model of craniosurgical intervention. Custom-made iPPG system was exploited to visualize intracranial vessels in anesthetized Wistar rats (n = 15). Video frames of rat’s cortex were recorded concurrently with systemic blood pressure, end-tidal CO_2_, and electrocardiogram. We found that injection of dorzolamide (carbonic-anhydrase inhibitor) significantly increased the blood-pulsations amplitude in all animals by 35 ± 19% (*p* < 0.001). Such an increase negatively correlated with significant decrease in end-tidal CO_2_ by 32 ± 7% (*p* < 0.001). It is noteworthy that the dorzolamide injection did not lead to significant changes in systemic blood pressure. Concluding, pulsations amplitude is a marker of the vascular tone that can be used to evaluate the functional cerebrovascular reserve. Imaging PPG is a simple and convenient method to assess cerebral blood flow, including during various neurosurgical interventions.

## Introduction

Evaluation of cerebral perfusion is important for patients with cerebrovascular disease to diagnose the hemodynamic significance of vascular lesions and determine the optimal treatment tactics for the patient^1–3^. The reserve of cerebral blood flow can be estimated by measuring cerebrovascular reactivity to external stimuli in the form of various pharmacological agents possessing vasoactive properties. These drugs include the acetazolamide (inhibitor of carbonic anhydrase), which causes an acidosis and a significant increase in brain perfusion due to dilatation of intracranial arteries^4–6^. Acetazolamide causes a decrease in the resistance of cerebral vessels, accompanied by an increase in CO_2_ level and corresponding decrease in pH in the blood^7,8^. This substance increases cerebral blood flow markedly in unaffected vessels, whereas in areas where blood is supplied by stenotic or malformed vessels, the flow either increases slightly or remains unchanged^9^. Most studies devoted to measurements of cerebral blood flow were carried out using diagnostic methods such as Magnetic Resonance Imaging^6,8^, single-photon or positron emission tomography^3,5^, and Transcranial Doppler ultrasonography^10^. As a rule, the goal of this kind of research is to assess the cerebral perfusion of the patient either to determine the further tactics of his treatment, or to refine the prognosis^3^. However, in the case of surgical revascularization, it is often necessary to assess the functional reserve of cerebral vessels, both to clarify the region of the lesion and to control the adequacy of surgical intervention. Currently, intraoperative blood flow studies are limited either to perfusion computed tomography or laser Doppler flowmetry, which provide information limited in accuracy and volume^11^. Moreover, they require either close contact with brain tissue or injection of radiopaque substances, which limits the multiplicity of procedures.

Optical methods are considered as very promising for noninvasive measurements of blood flow parameters. Among them, the methods based on video recordings of the exposed brain under different illumination are very promising because of their low cost and simplicity of implementation^12–15^. Laser speckle contrast imaging uses coherent light illumination and provides continuous monitoring of cerebral blood flow in two dimensions with high resolution^12,15^ but results of this technique are ambiguous in interpretation^16^ due to multiple light scattering in biological tissue. Other optical techniques reveal cerebral hemodynamics after processing videos recorded at incoherent illumination^13,14,17^. The method of optical intrinsic signals imaging associate changes in cortical blood flow with slowly varying changes in the recorded video^13,17^ considering the signal modulation at the heartbeat frequency as an artifact^18^. In contrast, the heartbeat related modulation is the primary source of information about cortex blood perfusion in imaging photoplethysmography (PPG) technique^14,19^. The PPG method is the base of the pulse-oximetry technique, which is widely used in clinics for measuring and monitoring the oxygen saturation in blood^20^. It was shown that the ratio of the pulsatile and non-pulsatile components of the PPG-signal in pulse oximeters operating in the red and infrared light can be used for estimation of peripheral perfusion^21,22^. However, since the pulse oximeter requires contact with biological tissue, it can hardly be used to evaluate perfusion of the cerebral cortex. We have shown recently that an advanced modality of the photoplethysmography, imaging PPG system operating at incoherent green illumination, allowed us to evaluate cerebral perfusion over wide area in a non-contact manner^14,19^. It was found that the amplitude of the pulsatile component of the PPG signal (APC) at green light is inversely related to cerebral vascular resistance, which was increasing in response to a decrease and decreasing in response to an increase in systemic arterial blood pressure (ABP)^14^. Such a behavior of APC is due to a cerebrovascular reflex aimed at maintaining the constancy of the volumetric velocity of cerebral blood flow in a changing blood pressure. We hypothesize that with invariable blood pressure, APC variation can be directly related to the dynamics of fluctuations in brain perfusion. Therefore, it can increase in response to a decrease in vascular resistance caused by various pharmacological substances, e.g., by an administration of a carbonic anhydrase inhibitor. The use of this kind of substances for study of intraoperative estimation of cerebrovascular functional reserve in the animal model is especially interesting because of specific mechanism of their action: creation of tissue acidosis, opening up perspectives of changing of the medication test for a technique of controlled respiratory acidosis by means of transient hypercapnia. In our experiments, we used dorzolamide as an intravenous form of a carbonic anhydrase inhibitor. Its potency and safety during systemic injections in rats were previously demonstrated^23^.

The aim of our study was to demonstrate the feasibility of application the imaging PPG technique to assess spatiotemporal changes in the tone of intracranial vessels. In our experiments, these changes were caused by dorzolamide-stimulated acidosis in animal model of craniosurgical intervention. The potential use of this method will be express-evaluation of the cerebrovascular reserve in different parts of the brain during neurosurgical operations.

## Methods

### Animals and ethics

All experiments were performed in accordance with the ethical guidelines of the International Association for the Study of Pain, the Directive 2010/63/EU of the European Parliament and of the Council on the protection of animals used for scientific purposes and reported in compliance with the ARRIVE guidelines. The study protocol was approved by the Institutional Animal Care and Use Committee of Pavlov First St. Petersburg State Medical University and the Pavlov Institute of Physiology of the Russian Academy of Sciences. All efforts were made to minimize animal suffering and to use only the number of experimental subjects necessary to produce reliable data.

Adult (mean body weight 409 ± 42 g, n = 15) male Wistar rats were purchased from the State Breeding Farm ‘‘Rappolovo” (Saint Petersburg, Russia). The animals were housed in groups (2–5 per cage) under standard laboratory conditions (12-h light/dark schedule) with food and water available ad libitum.

### Anesthesia and surgical preparation

Rats were anaesthetized by intraperitoneal injection with a mixture of urethane (Sigma, St. Louis, MO, USA) and a-chloralose (Sigma, St. Louis, MO, USA) at an initial dose of 800/60 mg/kg. Once a surgical level of anesthesia was achieved, the trachea was intubated for measurements of respiratory airflow and end-tidal carbon dioxide, the left femoral artery and vein were cannulated for continuous monitoring of blood pressure and drug administration, respectively. The animal’s head was fixed in a stereotaxic apparatus (Stoelting Co., Wood Dale, IL, USA) for preparation of a cranial window without violating the integrity of the cranial cavity^24^. To achieve this, the parietal bone was thinned using a micro-drill until the intracranial vessels were clearly visible through the remaining intact bone. During the drilling, tissues were cooled using topical application of cold saline. The surface of the closed cranial window was covered with mineral oil to improve the transparency of the residual bone for intravital video recordings of intracranial vessels. Steel needle electrodes were installed in the muscle tissue of rat paws to record the electrocardiogram (ECG) during the experiment. After surgical preparation, the rat rested for at least 40 min to minimize the effect of postsurgical reaction. Eight animals were paralyzed with pipecuronium bromide (0.9 mg/kg initially; maintained with 0.45 mg/kg as required, i.v.; “Arduan”, Gedeon Richter, Budapest, Hungary) and artificially ventilated with room air (SAR-830, CWE, Inc., Ardmore PA, USA).

**Figure 1:**
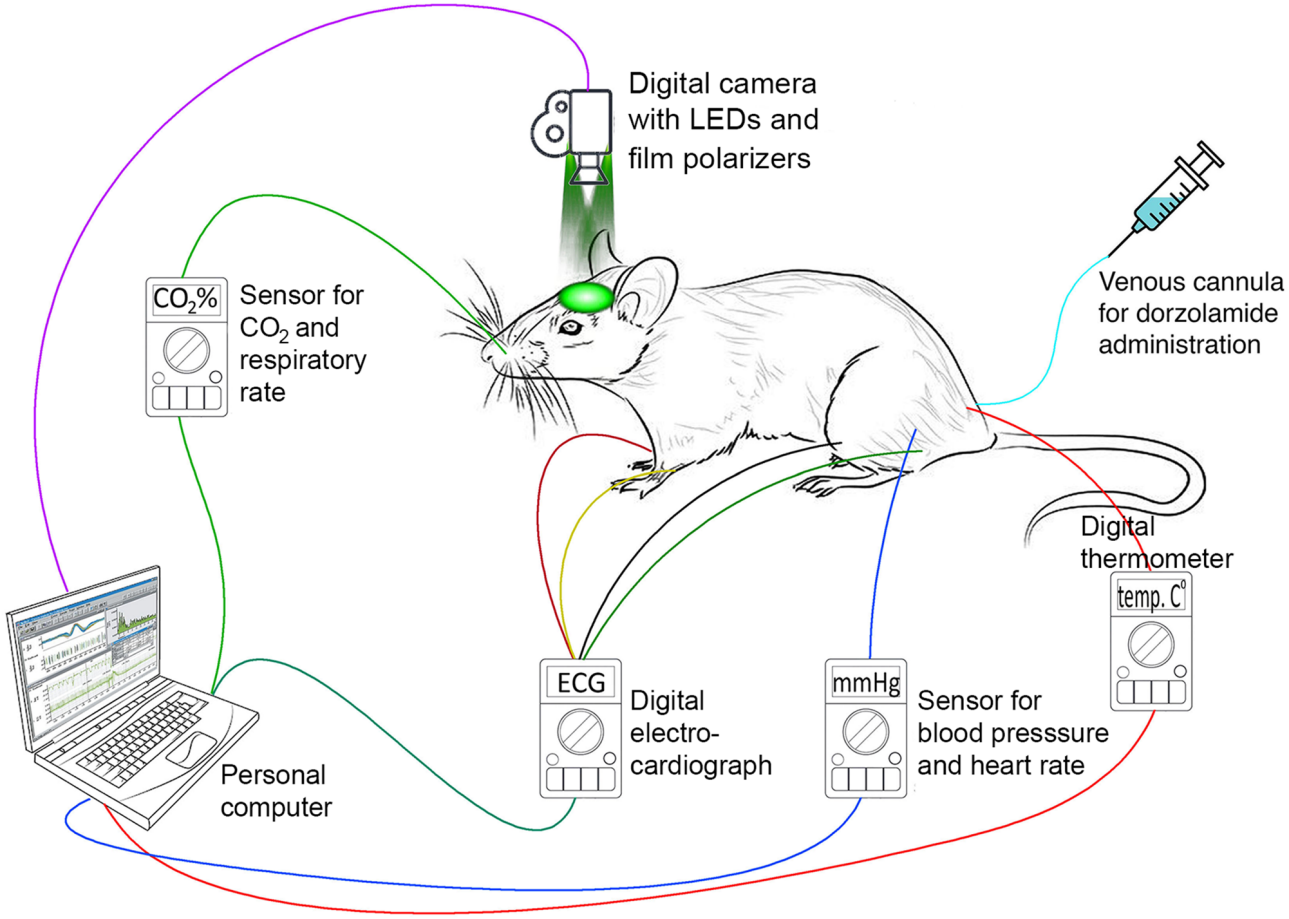
Layout of the experimental setup for monitoring the response of the cortical-blood-flow and key-physiological parameters to the dorzolamide administration.

Throughout all experiments, the rectal temperature was monitored and maintained within a range of 37–38 °C using a thermostatically controlled heating pad. ABP with the heart rate (HR) and end-tidal CO_2_ were continuously monitored by a pressure transducer (MLT844, AD Instruments Inc., Colorado Springs, USA) and Carbon Dioxide Analyzer (Capstar-100, CWE, Inc., Ardmore PA, USA), respectively. These data were digitized at the sample frequency of 10 kHz (ADC-DAC Power1401-3, Cambridge Electronic Design, Cambridge, UK) and recorded in the personal computer using Spike2 version 8 software (Cambridge Electronic Design, Cambridge, UK). Layout of the experimental setup with the positions of the sensors to monitor key physiological parameters of a rat is shown in Fig. 1. The adequacy of anesthesia was controlled by absence of withdrawal reflex after paw pinch (without myorelaxation) or severe (> 20%) blood pressure fluctuations (after myorelaxation). If necessary, a supplemental dose of the anaesthetic mixture of urethane/a-chloralose (75/5 mg/kg) was administered intravenously. After the end of each experiment, the rat was euthanized by intravenous injection of a lethal dose of urethane (3 g/kg).

### Experimental protocol

The total duration of each experiment was 360 s during which video frames of intracranial structures were recorded continuously and synchronously with ECG, HR, ABP, and end-tidal CO_2_. First, all these parameters of the rat in the baseline were recorded during the first 120 s. Second, the dorzolamide (“Dorzolan Solo”, Groteks, Ltd., St. Petersburg, Russia; 2 mg in 0.7 ml of saline) was injected into rat’s femoral vein in a slow rate during 60 s. Third, all the aforementioned parameters were continuously recorded both during the injection and for the next 180 s.

### Experimental arrangement

Parameters of cortical blood flow were measured by using custom-made imaging PPG system described in details in our previous papers^14,25^. Briefly, the system consisted of a digital monochrome camera with complementary metal–oxide–semiconductor sensing matrix (10-bit model GigE uEye UI-5220SE of the Imaging Development Systems GmbH) and illumination block. The latter contained eight light-emitting diodes (LEDs) mounted around the camera lens (25 mm focal length) in such a way as to ensure uniform illumination of the rat’s cortex through the cranial window. Incoherent light generated by LEDs at the wavelength of 530 ± 25 nm was linearly polarized by means of a film polarizer (Edmunds Optics, 0.18 mm thickness) attached to the LED assembling. To increase the signal-to-noise ratio, we used the polarization-filtration technique implemented in the form of another film polarizer (with orthogonal orientation to the first one) attached to the camera lens^26^. The imaging PPG system with LEDs and polarizers was located 15 cm above the rat’s cortex (see Fig. 1). The videos were recorded at 100 frames per second with resolution of 752 × 480 pixels and saved frame-by-frame in the hard disk of a personal computer.

### Data processing

**Figure 2:**
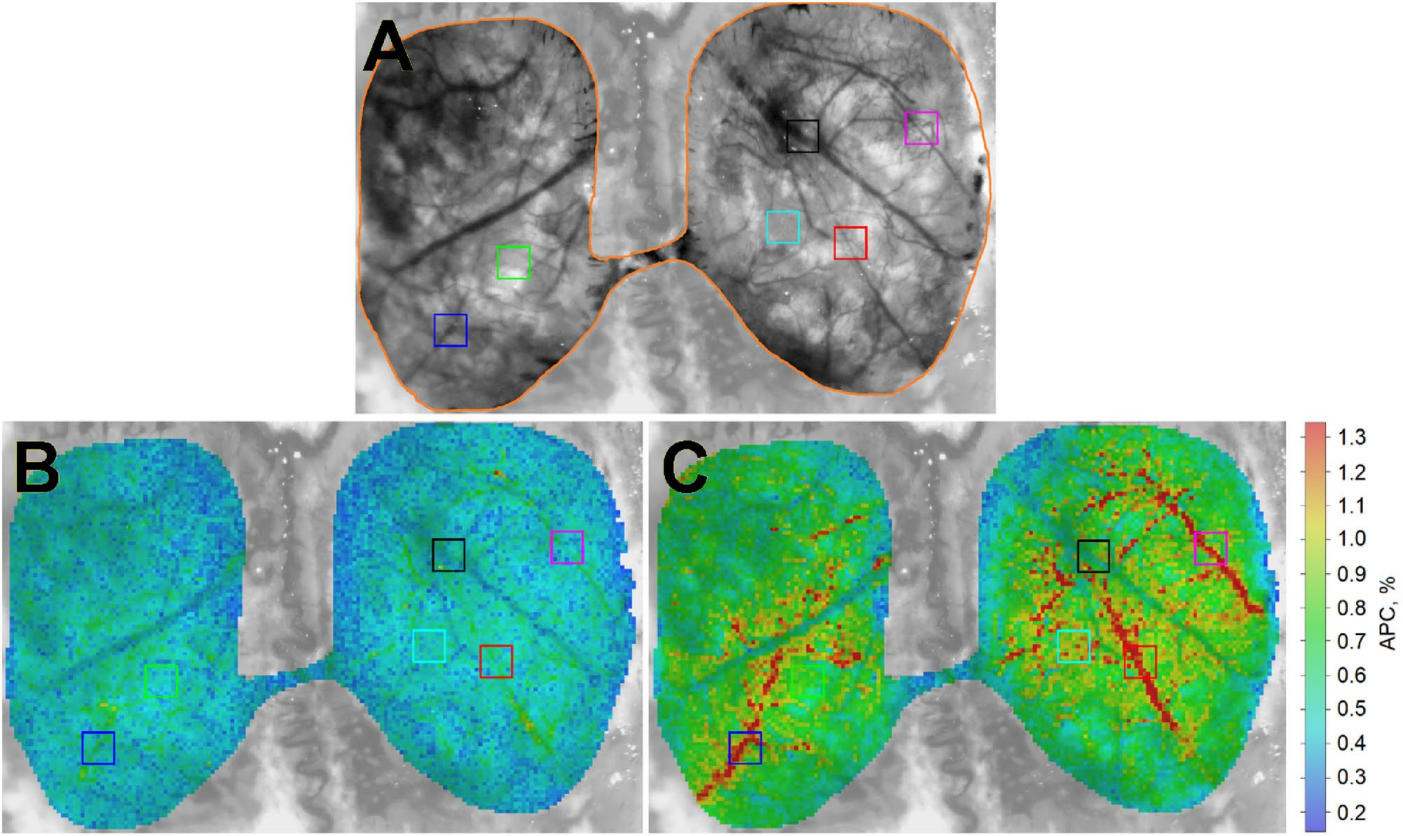
Spatial distribution of the amplitude (APC) of the optical signal oscillating at the heart rate for one of the rats. (**A**) One of the recorded frames of the rat’s cortex with a dedicated area (marked by the orange line) in which the photoplethysmographic waveform was assessed. (**B**) Spatial distribution of APC overlaid with the initial cortex image as estimated at the first second of the experiment. (**C**) Mapping of APC after the dorzolamide injection calculated at 340th second of the experiment. The color scale on the right shows APC in percent, which is the same for both panels (**B**) and (**C**). Colored squares show the position of the selected areas (big ROIs) in which changes in mean APC were estimated.

All recorded video frames, ECG, ABP, and CO_2_ data were processed off-line by using custom software implemented in the Matlab platform (Version, R2018b, The MathWorks, Inc., MA, USA, 2018). Recorded video frames were processed jointly with ECG by using an algorithm described in details in our previous papers^14,25^. The PPG waveform was calculated as frame-by-frame evolution of the mean pixel value in every small ROI sizing 3 × 3 pixels (which corresponds to the area of 65 × 65 μm^2^ at the cranial window) after spatial stabilization of the recorded frames^14^. Borders of every heart beat in the time scale were detected by using R-peaks of ECG recorded synchronously with video frames. Then, a mean PPG pulse was calculated by averaging the waveforms of 10 subsequent cardiac cycles defined by R–R intervals after normalizing the component oscillating at the heart rate to a slowly varying component of the PPG^25^. The mean PPG pulse was used to determine the parameter APC representing blood flow as difference between its maximum and minimum value. Depending on the rat’s HR, the time interval of 10 heartbeats varied from 1.3 to 2.2 s for the rats under study. Since APC was estimated in every small ROI, we were able to mapping this parameter over the rat’s cortex. To assess the dynamics of cerebral blood flow in response to dorzolamide injection, we calculated APC maps once every 10 s. Representative examples of calculated APC maps for one of the rats are shown in Fig. 2. The color-coded intensity maps in Fig. 2 give a visual impression of APC parameter distribution over rat’s cortex. While the map in Fig. 2B was calculated at the baseline (1-st second of the experiment), that in Fig. 2C shows the APC distribution calculated at 340 s (160 s after the end of dorzolamide injection). As seen in Fig. 2C, pulsating arteries are clearly distinguished from the veins because the arteries have much higher amplitude of the pulsatile component synchronized with cardiac activity. Comparing the maps in panels B and C, a significant increase in APC across the whole cortex is clearly visible.

For quantitative analysis of the cortical blood flow changes in response to dorzolamide injection, we selected six big ROIs of 27 × 27 pixels sizing 0.59 × 0.59 mm^2^ at the rat’s cortex. To check whether there is a difference in the dynamics of changes in the APC parameter in different areas of the cerebral cortex, big ROIs were situated in areas with different blood vessels. In the example shown in Fig. 2, red, blue and magenta ROIs were selected in the area of large arteries, black ROIs in the area of large vein, and two ROIs (green and cyan) were placed between large vessels in the observation plane. Averaging APC over all small ROIs within a large ROI for each distribution calculated every 10 s, we obtained the APC evolution in selected big ROIs.

One can clearly see in Fig. 2B,C that spatial distribution of APC is highly inhomogeneous. Even in the baseline (Fig. 2B), the mean APC in blue ROI near an artery is much higher than that in black ROI situated at a vein: 0.8% versus 0.3%. To compensate for the initially spatially inhomogeneous distribution of APC, determined by the geometry and morphology of the cortical vessels, we normalized the current value of APC in each big ROI to its average value during the baseline (first 120 s of recording). This normalized parameter is denoted as APC_N_.

### Statistical analysis

In each of the 15 experiments, the physiological indices were evaluated initially as an average value for 120 s and then the averaging was done for 180 s after finishing the dorzolamide administration. To estimate the difference in the measured indicators before and after drug administration, we used the nonparametric paired difference tests (sign and Wilcoxon sign-rank test). To assess the relationship between the dynamics of the indicators, Pearson correlation was used. The level of *p* < 0.05 was considered as significant. The average value of the correlation coefficient was calculated to demonstrate the pattern of parameters correlation in the studied animal population. The statistical analysis of the measured data was carried out by using the software Statistica 10 (StatSoft, Russia).

## Results

### Mapping the amplitude of blood pulsations

**Figure 3:**
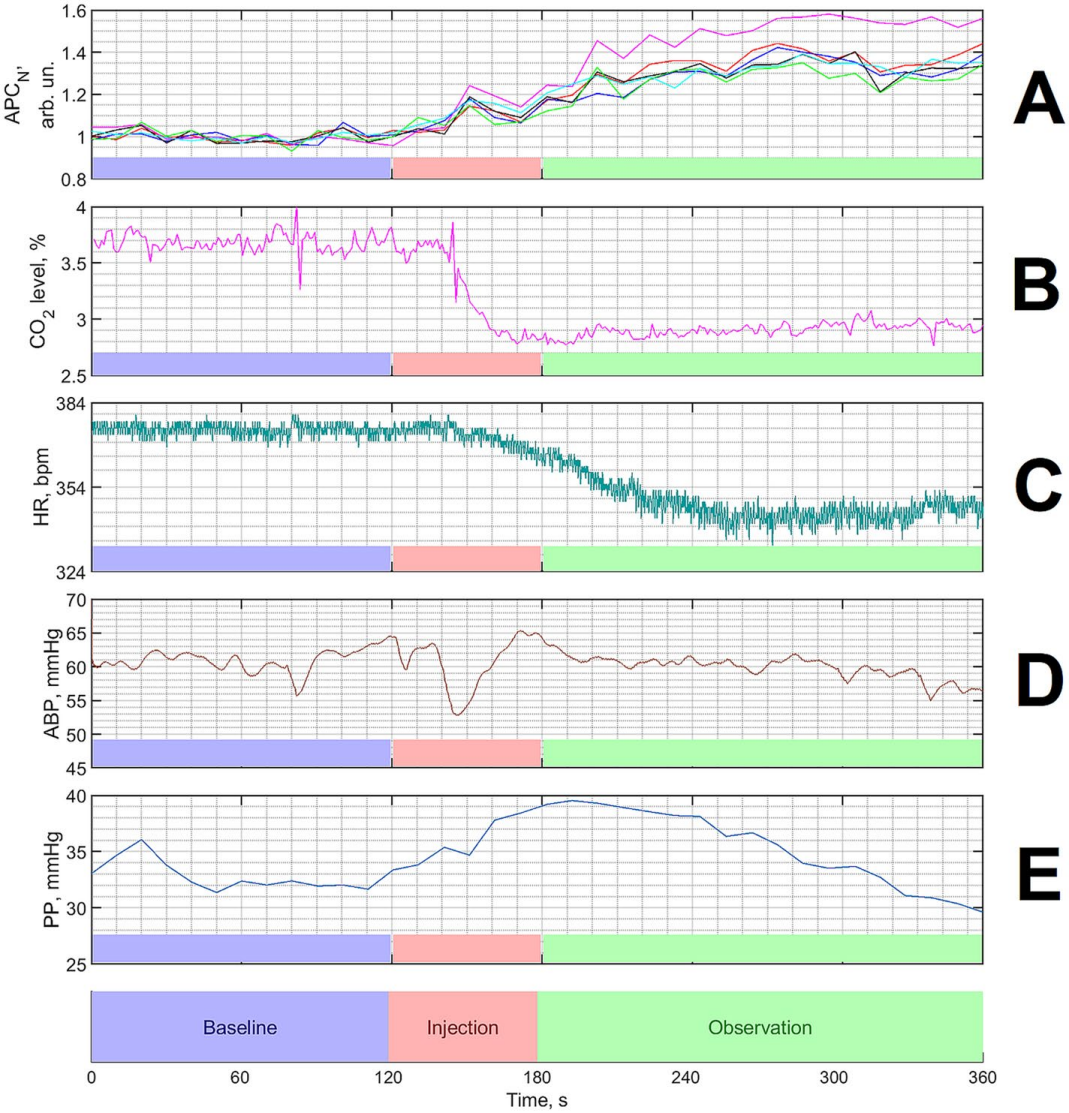
Typical responses of the measured physiological indices on the dorzolamide injection for one of the rats. (**A**) The normalized amplitude of the pulsatile component (APC) of the photoplethysmographic waveform assessed in the six selected areas shown in Fig. 2. The color of the waveforms coincides with that of the squares in Fig. 2 indicating the position of the big areas of interest. (**B**) Concentration of end-tidal carbon dioxide (CO_2_). (**C**) Dynamics of the heart rate. (**D**) Systemic arterial pressure (its mean value, ABP). (**E**) Pulse pressure (PP). ABP and PP were measured invasively through a cannula in the animal’s femoral artery. The timeline (which is the same for all graphs) is displayed at the bottom indicating the stages of the experimental protocol.

In our experiments, blood microcirculation of the cerebral cortex was assessed by imaging PPG technique at green light successfully for all the animals under study. Spatial distribution of the APC parameter characterizing cortical blood perfusion was estimated over the entire cortex through the residual bone of the rat skull. A significant increase in APC after dorzolamide injection was found in each rat, as illustrated in an example shown in Fig. 2B,C. Spatiotemporal dynamics of changes in APC maps recorded once every ten seconds for one of the rats is shown in Supplementary Movie online. It is worth noting that for all rats in spite of initially inhomogeneous distribution of APC, the normalized parameter APC_N_ estimated in big ROIs selected in different areas of the cortex (such as arteries, veins or between them) showed a similar response to dorzolamide injection. This similarity can be seen in Fig. 3A in which a typical evolution of the APC_N_ parameter estimated in the selected big ROIs before, during, and after dorzolamide injection is presented. The observed dynamics of APC_N_ suggests a systemic response of intracranial vessels to the dorzolamide injection.

### The response of physiological indices to the dorzolamide injection

Figure 3 shows the APC_N_ dynamics (measured in the contactless manner) together with four physiological indices (end-tidal CO_2_, HR, mean ABP, and pulse pressure, PP, all measured invasively) for one of the rats. As seen, the drug injection leads to a significant increase in APC_N_ accompanied by a decrease in both CO_2_ concentration and heart rate, whereas reaction of the mean ABP is stochastic. Similar changes of the physiological indices due to the drug injection were observed in all 15 rats under the study (see responses of the indices on dorzolamide administration in every rat in Supplementary Fig. S1 through Fig. S15 online).

**Figure 4:**
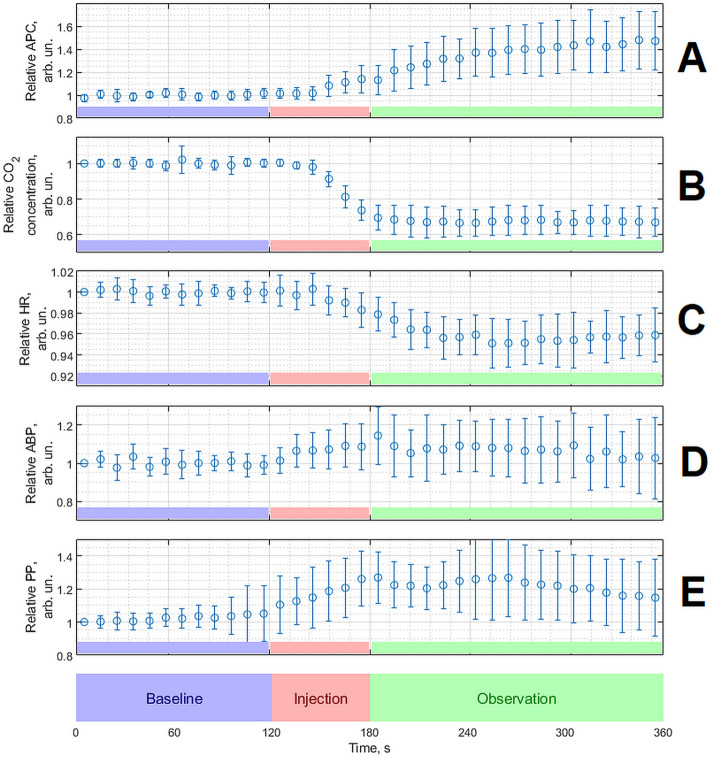
Response of the physiological parameters averaged over the whole group (n = 15) of animals to the dorzolamide injection. (**A**) Normalized amplitude of the pulsatile component of (APC_N_) the photoplethysmographic waveforms. (**B**) Relative changes in the end-tidal carbon dioxide (CO_2_) concentration. (**C**) Relative changes in the heart rate (HR). (**D**) Relative changes in the mean systemic arterial blood pressure (ABP). (**E**) Relative changes in the pulse pressure (PP) measured invasively in femoral vein. The circles in all graphs show the mean value whereas the whiskers are standard deviation. In all panels, the data before averaging were normalized to the respective mean value during the baseline for the correctness of averaging the experimental results with different rats.

To compare the dorzolamide effect among different animals, we averaged the values of the indices for each rat during the baseline (0–120 s of recording) and normalized every parameter to its respective mean value in the baseline. The initial values of the averaged physiological indices during the baseline varied from one rat to another: 2.87 ± 0.41 (2.15–3.96)% of the end-tidal CO_2_ concentration, 396 ± 46 (287–485) bpm of the heart rate, and 60 ± 10 (40–87) mmHg of the mean systemic blood pressure. Evolution of the normalized parameters of APC_N_, CO_2_, HR, mean ABP, and PP for all animals is shown in Fig. 4 where the circles are the particular parameter averaged over the whole sample, and the bars are the standard deviation.

It is clearly seen in Fig. 4 that a change in all indices except for the mean level of blood pressure in response to dorzolamide injection was visually significant for all rats. For a quantitative estimation of the drug-injection influence, we calculated the difference between the mean values of every physiological index during the period of observation (180–360 s) and the baseline for each rat. We show these data in Table 1 for every animal under study as normalized to the mean respective index during the baseline (in percent).

**Table 1:** Relative changes in the physiological indices of APC, end-tidal CO_2_, heart rate, mean blood pressure (ABP), and pulse pressure (PP) after administration of dorzolamide in the animals under study.

Rat #	ΔAPC, %	ΔCO_2_, %	ΔHR, %	ΔABP, %	ΔPP, %
1	72	− 36	− 6.4	− 7.3	7.8
2	32	− 37	− 2.5	27.7	6.8
3	15	− 37	− 3.8	0.0	11.3
4	46	− 34	− 3.1	− 12.5	28.1
5	23	− 23	− 2.8	4.3	15.2
6	40	− 30	− 5.6	14.8	49.7
7	50	− 37	− 3.9	− 10.0	6.2
8	34	− 23	− 0.5	41.3	33.4
9	11	− 37	− 3.9	10.1	5.8
10	13	− 33	− 4.7	9.4	29.4
11	37	− 21	− 7.1	− 1.6	16.8
12	40	− 31	− 5.8	7.8	22.0
13	55	− 43	− 4.8	− 6.5	6.8
14	59	− 24	− 3.7	13.6	36.8
15	19	− 39	− 3.9	− 6.4	6.4
Mean ± SD	36 ± 17	− 32 ± 7	− 4.2 ± 1.6	5.47 ± 14.3	18.8 ± 13.8

As seen, a decrease in the end-tidal CO_2_ level was observed in all animals with the mean over the group of − 32 ± 7%, *p* < 0.001. These observations indicate the effectiveness of the direct effect of the carbonic anhydrase inhibitor that leads to a delay of CO_2_ in the body because of a decrease in the dissociation of carbonic acid in tissues and red blood cells. Against this background, dorzolamide injection led to a significant increase in the optically measured APC-parameter in all laboratory animals: its value averaged over the whole group increased by 36 ± 17%, *p* < 0.001. The increase in APC was accompanied by a decrease in HR, which decreased in all rats after injection. On average, the decrease in the group was − 4.2 ± 1.6%, *p* < 0.001. In contrast, the change in the mean ABP were both positive and negative: slight increase was observed in nine rats whereas the ABP decreased in six animals. One rat showed no change in systemic blood pressure. The mean group value of ABP did not reveal statistically significant changes in response to dorzolamide injection: 5.7 ± 14.3%, *p* = 0.09, although there was a tendency to ABP growth. However, the pulse pressure (PP) shows statistically significant increase after dorzolamide injection: 18.8 ± 13.8%, *p* < 0.001.

### Relationship between the dynamics of physiological indices

To reveal the relationship between the analyzed physiological parameters (APC, end-tidal CO_2_, HR, ABP, and PP), cross-correlations between each pair of them were calculated by using the data measured once every ten seconds (an example is shown in Fig. 2) for all animals under study. The results of the correlation analysis are presented in Table 2. The mean value of the correlation coefficient presented in the last row of Table 2 demonstrates the correlation strength between the parameters in the studied sample of the rats. It is clearly seen that three physiological parameters (APC, end-tidal CO_2_, and HR) are related each other by a strong correlation: for most animals, *p* < 0.001. Our experiments showed that decrease in CO_2_ concentration in all rats correlated with the increase in APC (negative correlation) resulting in the group average correlation coefficient of − 0.80. Moreover, the increase in APC in all animals was associated with a decrease in heart rate (negative correlation, as well), with the mean correlation coefficient for the whole group of − 0.79. In turn, the dynamics of HR in all (except rat #8) was directly related to the dynamics of end-tidal CO_2_ concentration (positive correlation with the mean r = 0.79). At the same time, either changes in mean ABP did not correlate with changes in other physiological indices, or the correlation was of different sign in different animals (see columns 5–7 in Table 2). These observations indicate absence of a systemic effect of dorzolamide injection on the changes in the mean ABP consistently with changes of other measurable physiological indices (APC, CO_2_, and HR).

**Table 2:** Correlation coefficients between the pairs of the physiological parameters APC_N_, CO_2_, HR, mean systemic ABP, and systemic PP measured in 15 rats.

Rat	APC_N_ vs. CO_2_	APC_N_ vs. HR	CO_2_ vs. HR	APC_N_ vs. ABP	CO_2_ vs. ABP	HR vs. ABP	APC_N_ vs. PP	CO_2_ vs. PP	HR vs. PP	ABP vs. PP
1	− 0.75*	− 0.93	0.74	− 0.70	0.26 *p* = 0.13	0.74	− 0.15 *p* = 0.37	− 0.45	0.16 *p* = 0.36	0.64
2	− 0.74	− 0.78	0.75	0.42	− 0.70	− 0.18 *p* = 0.28	− 0.06 *p* = 0.74	− 0.30 *p* = 0.08	0.06 *p* = 0.71	0.43
3	− 0.65	− 0.88	0.77	− 0.49	− 0.06 *p* = 0.74	0.49	0.36	− 0.87	− 0.50	0.40
4	− 0.78	− 0.82	0.91	0.41	− 0.38	− 0.37	0.91	− 0.82	− 0.88	0.52
5	− 0.82	− 0.89	0.82	0.36	− 0.33 *p* = 0.05	− 0.16 *p* = 0.34	0.77	− 0.83	− 0.81	0.48
6	− 0.91	− 0.91	0.87	0.69	− 0.77	− 0.69	0.89	− 0.95	− 0.90	0.85
7	− 0.88	− 0.76	0.85	− 0.54	0.71	0.75	0.47	− 0.38	− 0.43	− 0.07 *p* = 0.68
8	− 0.81	− 0.50	0.16 *p* = 0.35	0.64	− 0.67	0.19 *p* = 0.26	0.43	− 0.44	0.30 *p* = 0.07	0.67
9	− 0.74	− 0.63	0.92	− 0.05 *p* = 0.75	− 0.22 *p* = 0.20	− 0.31 *p* = 0.07	− 0.22 *p* = 0.19	− 0.16 *p* = 0.34	− 0.30 *p* = 0.07	0.87
10	− 0.72	− 0.83	0.91	0.31 *p* = 0.07	− 0.46	− 0.35	0.59	− 0.77	− 0.68	0.78
11	− 0.86	− 0.96	0.79	− 0.27 *p* = 0.12	0.11 *p* = 0.51	0.17 *p* = 0.33	0.74	− 0.84	− 0.74	0.19 *p* = 0.27
12	− 0.87	− 0.80	0.82	0.12 *p* = 0.50	− 0.28 *p* = 0.10	− 0.23 *p* = 0.17	0.68	− 0.85	− 0.73	0.53
13	− 0.96	− 0.91	0.91	− 0.73	0.65	0.57	0.20 *p* = 0.25	− 0.30 *p* = 0.08	− 0.37	0.34
14	− 0.94	− 0.85	0.79	0.36	− 0.52	− 0.15 *p* = 0.37	0.79	− 0.89	− 0.52	0.80
15	− 0.87	− 0.76	0.91	− 0.44	0.32 *p* = 0.06	0.51	− 0.11 *p* = 0.54	− 0.21 *p* = 0.21	− 0.19 *p* = 0.27	0.61
Mean r	− 0.82	− 0.81	0.79	0.01	− 0.16	0.07	0.42	− 0.60	− 0.44	0.54

*For all values given alone, *p* < 0.05.

Despite statistically significant increase in mean pulse pressure for the whole group after dorzolamide injection, no correlation between APC_N_ and PP was found for 5 of 15 rats (see column 8 in Table 2). Moreover, we observed high variability in the dynamics of the PP response to the injection. First type of the response, a sharp increase in PP during injection accompanied by an equally sharp decline immediately after its completion, was observed in rats # 3, 10, 13, 15 (see Supplementary Figs. S3, S10, S13, and S15), No decrease in PP after completion the dorzolamide injection was observed in the second group (rats # 6, 9, 12, 14, Supplementary Figs. S6, S9, S12, and S14), while there is no correlation between changes in PP and moments of injections in the third group (rats # 4, 7, 8, Supplementary Figs. S4, S7, and S8). In contrast, there is no principle difference in the dynamics of the APC response: it is always increasing during and after injection in all 15 rats (see Supplementary Figs. S1–S15 online). It should be emphasized that PP was assessed at the level of the femoral artery, thus reflecting changes in systemic hemodynamics. On the contrary, APC was measured locally at the cortex reflecting local changes in the intracranial blood flow. Since the measuring sites are different, changes to these two parameters are not necessarily related. Therefore, after the administration of a carbonic anhydrase inhibitor, dorzolamide, a significant increase in APC is observed, which is not caused by variations of the systemic blood pressure. Considering significant changes in the index APC, which characterizes cortical blood perfusion, due to dorzolamide injection, this parameter can be used to assess the functional reserve of cerebral vessels providing conditions for insignificant variations in the systemic blood pressure.

## Discussion

We demonstrated for the first time that imaging PPG technique made it possible to visualize cerebral blood flow and its variations through a thinned bone, without removing the dura mater and even without violating the integrity of the cranial cavity in all 15 rats under study. It was found that intravenous injection of the carbonic anhydrase inhibitor, dorzolamide, led to a significant decrease in the concentration of end-tidal CO_2_. This observation indicates a direct effect of the drug consisting of suppression the dissociation of carbonic acid in the tissue and erythrocytes^27^, which is developing within a minute after injection. As known, the main consequence of carbonic acid accumulation is tissue acidosis, which reduces the tone of the cerebral vessels. In our experiment, we have observed significant increase in the APC of the cerebral cortex in all animals. As it was recently shown, an increase of cortical APC is related to the decrease of the vascular tone^14^. Experiments carried out in the cited paper demonstrated that changes in cortical APC are in the counter-phase with variations in the systemic ABP. As suggested, such a relationship between APC and ABP is likely due to the cerebrovascular reflex aimed at maintaining the perfusion of brain tissue in a stable level^14^.

One could assume that tissue acidosis could lead to a decrease in systemic mean ABP. However, in our experiments, instead of diminishing mean ABP by drug injection, a tendency to its increase was revealed accompanied by a simultaneous significant increase in APC. Changes in the mean ABP were not systemically associated either with changes in end-tidal CO_2_ concentration or with changes in APC: in different animals, there was both positive and negative correlation between mean ABP and any of these indices, whereas no correlation between these parameters was observed in several rats (see Table 2). It is worth noting that dorzolamide injection leads to an increase in PP measured in the femoral artery (Table 1). However, since PP and APC were measured in different sites, their changes are not necessarily related. We assume that the increase in APC may be due to an increase in PP, which can grow in response to a decrease in arteriole tone in conditions of constant systemic blood pressure. The task of the dorzolamide test is to assess the degree of decrease in vascular tone of the intracranial vessels, which is characterized by the degree of increase in APC measured at the cortex. Therefore, significant increase in APC caused by intravenous injection of dorzolamide indicates a decrease in vascular tone regardless of the dynamics of the systemic mean ABP. As known, a decrease in the vascular tone under conditions of the constant ABP leads to an increase in regional cerebral blood flow^28,29^. In this regards, the observed decrease in HR most likely occurs reflexively in response to an increase in intracranial blood pressure^30^. In other words, administration of the carbonic anhydrase inhibitor leads to increased cerebral blood flow (in conditions of constant ABP), which is associated with a decrease in the tone of intracranial vessels in response to acidosis, and it is manifested by an increase in APC.

To summarize, we have shown that spatially resolved variations in time of the heart-related pulsations amplitude in cerebral vessels quantified by the parameter APC in contactless imaging PPG technique is a marker of the vascular tone. Under conditions of constant systemic blood pressure, APC can be used to assess the functional reactivity of blood vessels in response to acidosis caused by an administration of the carbonic anhydrase inhibitor. Since the imaging PPG method is quite simple to implement and allows data processing in real time, it is a convenient tool for assessing functional cerebrovascular reserve in intraoperative conditions, when the use of other techniques is difficult to implement. Note that big difference in brain size between rats and humans does not limit the high prospects for imaging PPG-system applications for intraoperative control of the cortex hemodynamics. Since this system provides measurements in the full field of view defined by a digital camera, the area under study can be readily chosen and adjusted by using different lenses and/or sensing matrices. An example of successful application of green-light imaging PPG system for blood-flow monitoring in human brain was recently published^31^.

In our functional test, vessels vasodilation was a consequence of acidosis caused by dorzolamide administration. Nevertheless, acidosis can also be induced by increasing the concentration of CO_2_ in inspired air providing possibility to carry out similar functional test in conditions of artificial pulmonary ventilation without administration of a carbonic anhydrase inhibitor. To create respiratory acidosis, it is enough to increase the concentration of CO_2_ in the respiratory mixture for a short period (e.g., using carbogen) or change the mode of artificial pulmonary ventilation. The proposed functional test creates wide prospects for the intraoperative assessment of the functional reserve of blood flow in the clinic during reconstructive neurosurgical operations in patients with cerebral arteriosclerosis or in open interventions after the stroke, making it possible to evaluate in real time the effectiveness of the surgical treatment itself.

## Supplementary information

Supplementary Video 1.

Supplementary Figures.
