# Author Correction: Animal model of assessing cerebrovascular functional reserve by imaging photoplethysmography

**DOI:** 10.1038/s41598-021-88330-4

**Published:** 2021-04-26

**Authors:** Oleg V. Mamontov, Alexey Y. Sokolov, Maxim A. Volynsky, Anastasija V. Osipchuk, Valery V. Zaytsev, Roman V. Romashko, Alexei A. Kamshilin

**Affiliations:** 1 Department of Circulation Physiology, Almazov National Medical Research Centre, Saint Petersburg, Russia; 2 Department of Departmental Therapy, Pavlov First Saint Petersburg State Medical University, Saint Petersburg, Russia; 3 Department of Neuropharmacology, Valdman Institute of Pharmacology, Pavlov First Saint Petersburg State Medical University, Saint Petersburg, Russia; 4 Pavlov Institute of Physiology of the Russian Academy of Sciences, Saint Petersburg, Russia; 5 Faculty of Applied Optics, ITMO University, Saint Petersburg, Russia; 6 Laboratory of High‑Precision Optical Measurements, Institute of Automation and Control Processes FEB RAS, Vladivostok, Russia; 7 Faculty of Photonics and Optical Information, ITMO University, Saint Petersburg, Russia

Correction to: *Scientific Reports* 10.1038/s41598-020-75824-w, published online 04 November 2020

The original version of this Article contained an error in the Funding section.

“This study was supported by the Ministry of Science and Higher Education of the Russian Federation, grant No. 075-15-2020-80.”

now reads:

“This study was supported by the Ministry of Science and Higher Education of the Russian Federation, grant No. 075-15-2020-800.”

The original Article has been corrected.

