# Supplementary material for: Animal model of assessing cerebrovascular functional reserve by imaging photoplethysmography
Source: Sci Rep. 2020 Nov 4;10:19008. doi: 10.1038/s41598-020-75824-w (PMC7642404; doi:10.1038/s41598-020-75824-w)
Supplement: Supplementary file 2 — Supplementary Figures. [file 41598_2020_75824_MOESM2_ESM.docx]

**Animal model of assessing cerebrovascular functional reserve by imaging photoplethysmography**

Oleg V. Mamontov^1,2^, Alexey Y. Sokolov^3,4^, Maxim A. Volynsky^5^, Anastasija V. Osipchuk^3,4^, Valery V. Zaytsev^5^, Roman V. Romashko^6^, and Alexei A. Kamshilin^5*^

^1^Department of Circulation Physiology, Almazov National Medical Research Centre, Saint Petersburg, Russia

^2^Department of Departmental Therapy, Pavlov First Saint Petersburg State Medical University, Saint Petersburg, Russia

^3^Department of Neuropharmacology, Valdman Institute of Pharmacology, Pavlov First Saint Petersburg State Medical University, Saint Petersburg, Russia

^4^Pavlov Institute of Physiology of the Russian Academy of Sciences, Saint Petersburg, Russia

^5^Faculty of Applied Optics, ITMO University, Saint Petersburg, Russia

^6^Laboratory of High-Precision Optical Measurements, Institute of Automation and Control Processes FEB RAS, Vladivostok, Russia

* Corresponding author e-mail: [alexei.kamshilin@yandex.ru](mailto:alexei.kamshilin@yandex.ru)

**SUPPLEMENTARY INFORMATION**


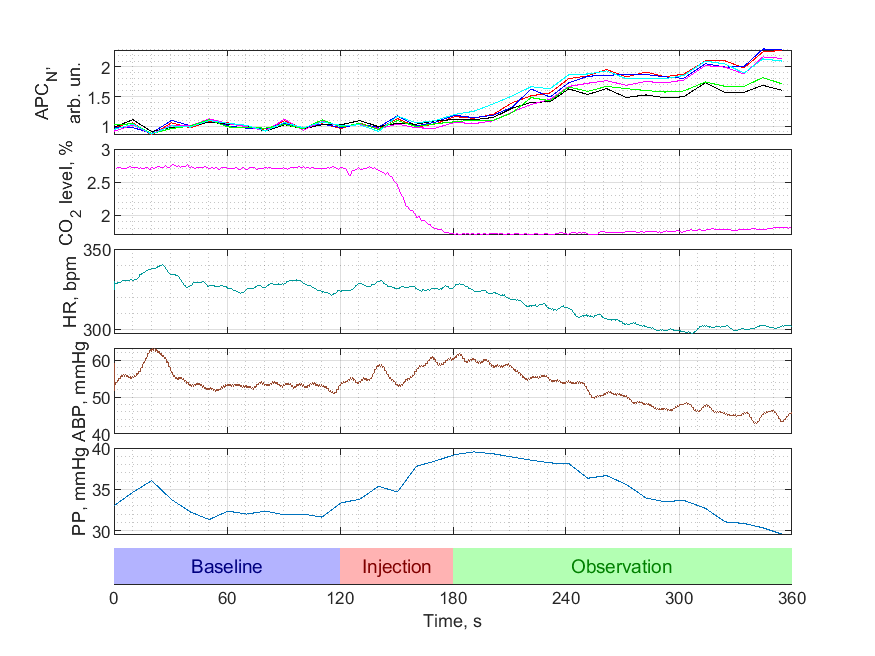


**Figure S1.** Responses of the measured physiological indices on the dorzolamide injection for the Rat #1 (measured at 22/05/2019, weight: 330 g)


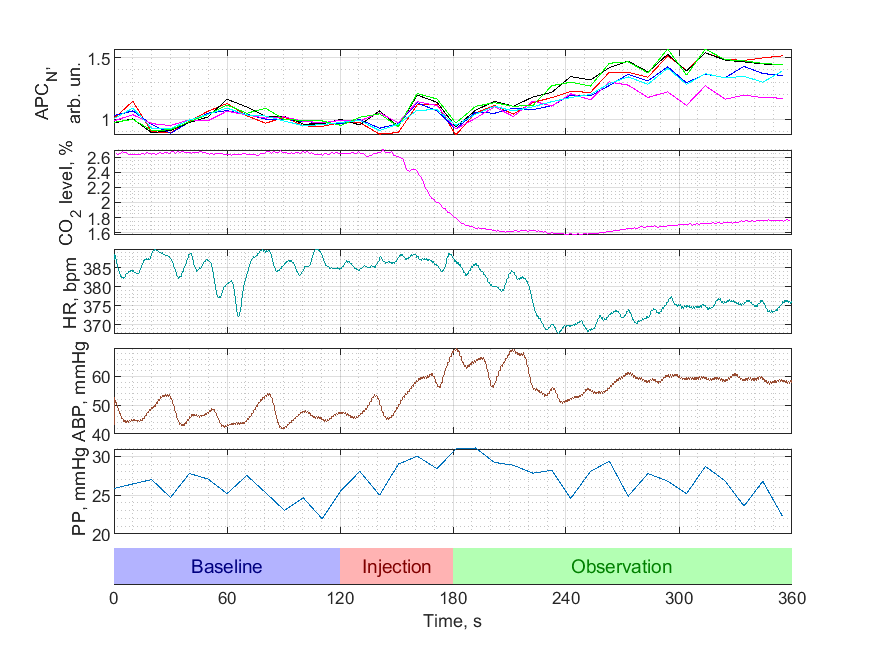


**Figure S2.** Responses of the measured physiological indices on the dorzolamide injection for the Rat #2 (measured at 29/05/2019, weight: 350 g)


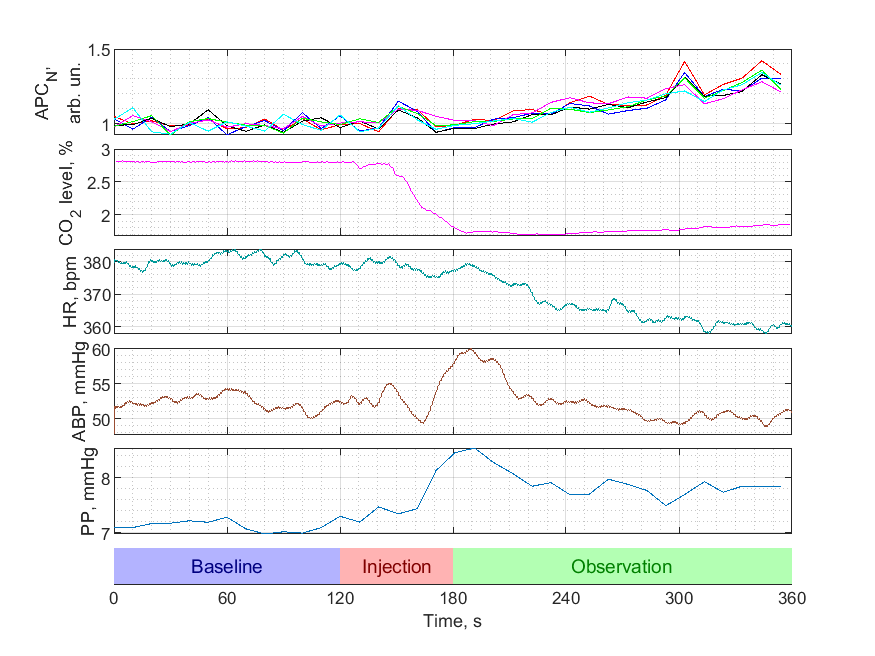


**Figure S3.** Responses of the measured physiological indices on the dorzolamide injection for the Rat #3 (measured at 11/06/2019, weight: 360 g)


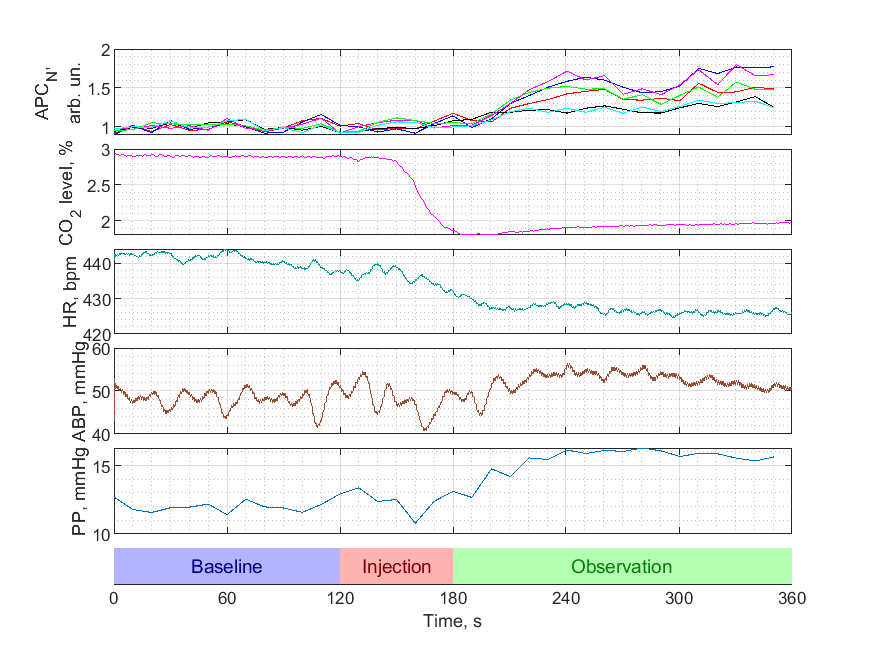


**Figure S4.** Responses of the measured physiological indices on the dorzolamide injection for the Rat #4 (measured at 19/06/2019, weight: 455 g)


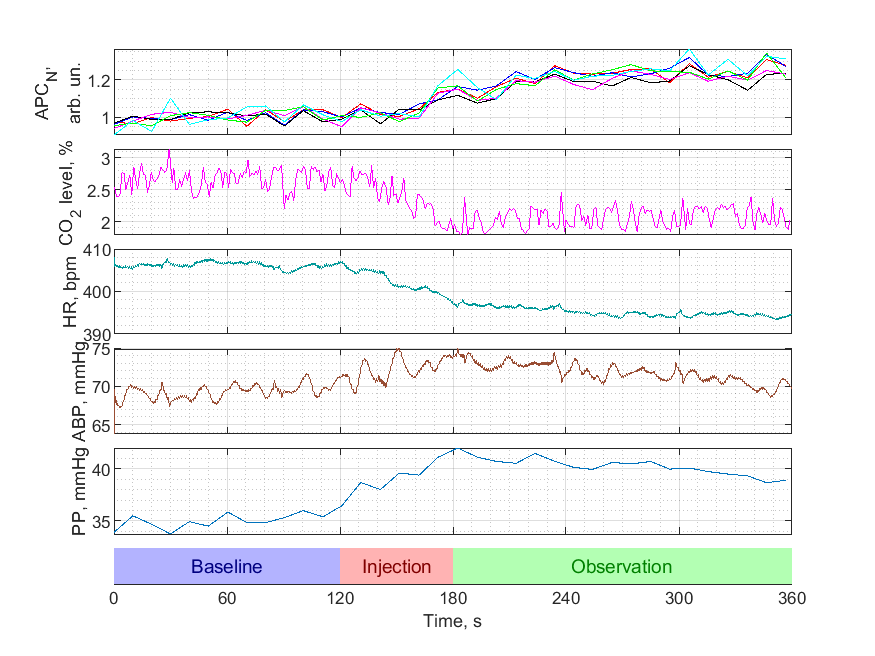


**Figure S5.** Responses of the measured physiological indices on the dorzolamide injection for the Rat #5 (measured at 03/07/2019, weight: 420 g)


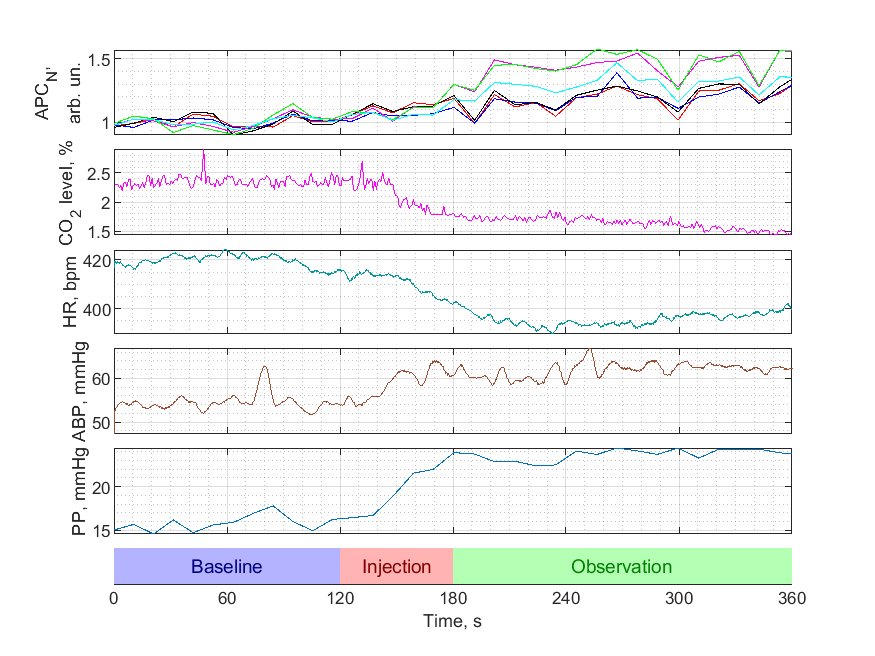


**Figure S6.** Responses of the measured physiological indices on the dorzolamide injection for the Rat #6 (measured at 08/07/2019, weight: 365 g)


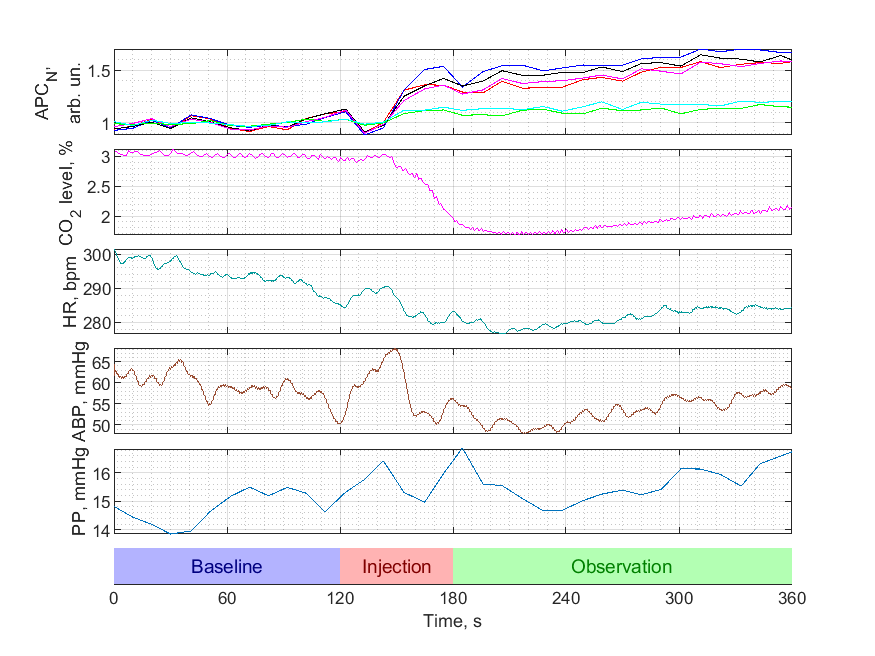


**Figure S7.** Responses of the measured physiological indices on the dorzolamide injection for the Rat #7 (measured at 10/07/2019, weight: 416 g)


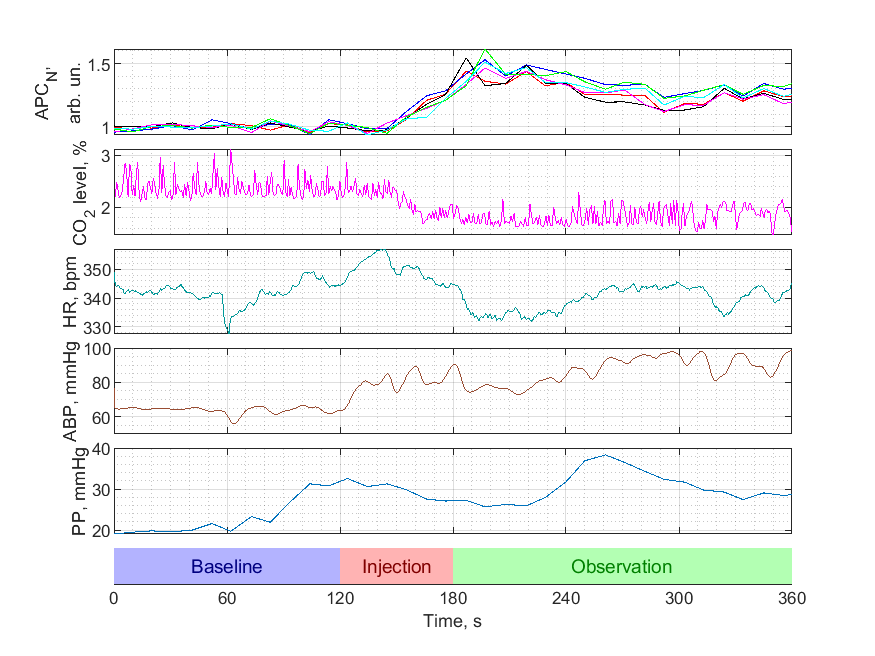


**Figure S8.** Responses of the measured physiological indices on the dorzolamide injection for the Rat #8 (measured at 17/07/2019, weight: 485 g)


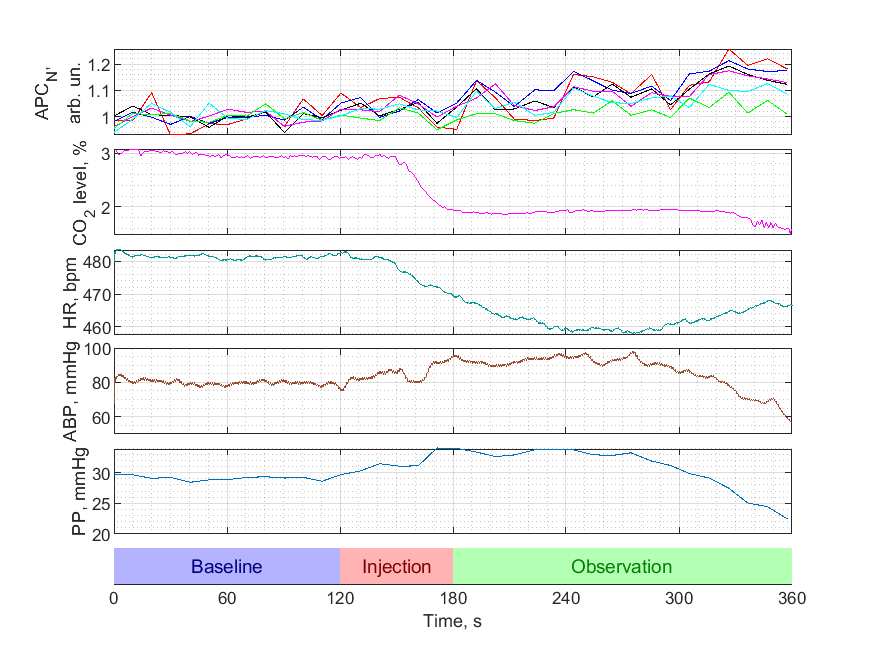


**Figure S9.** Responses of the measured physiological indices on the dorzolamide injection for the Rat #9 (measured at 22/07/2019, weight: 420 g)


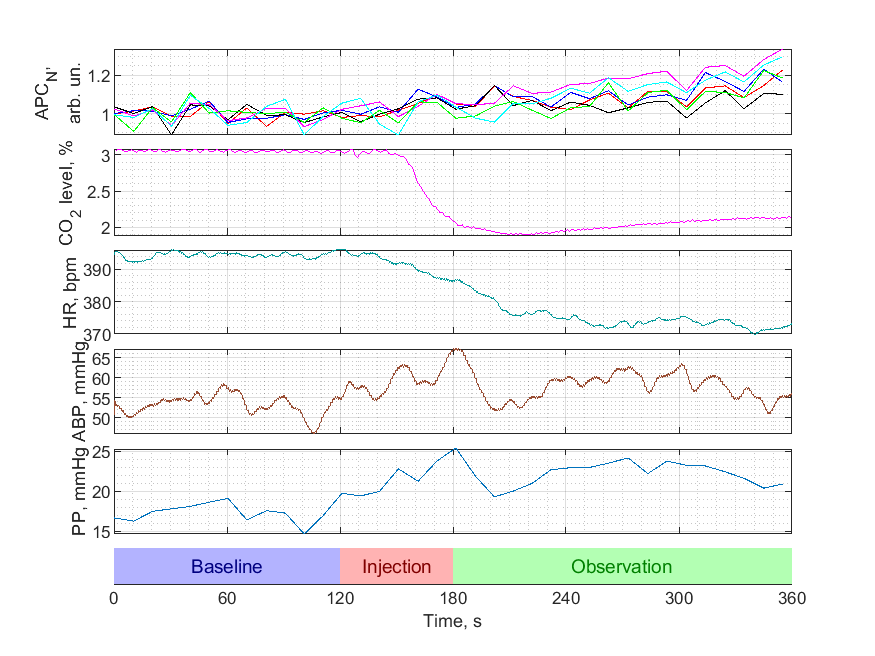


**Figure S10.** Responses of the measured physiological indices on the dorzolamide injection for the Rat #10 (measured at 25/07/2019, weight: 436 g)


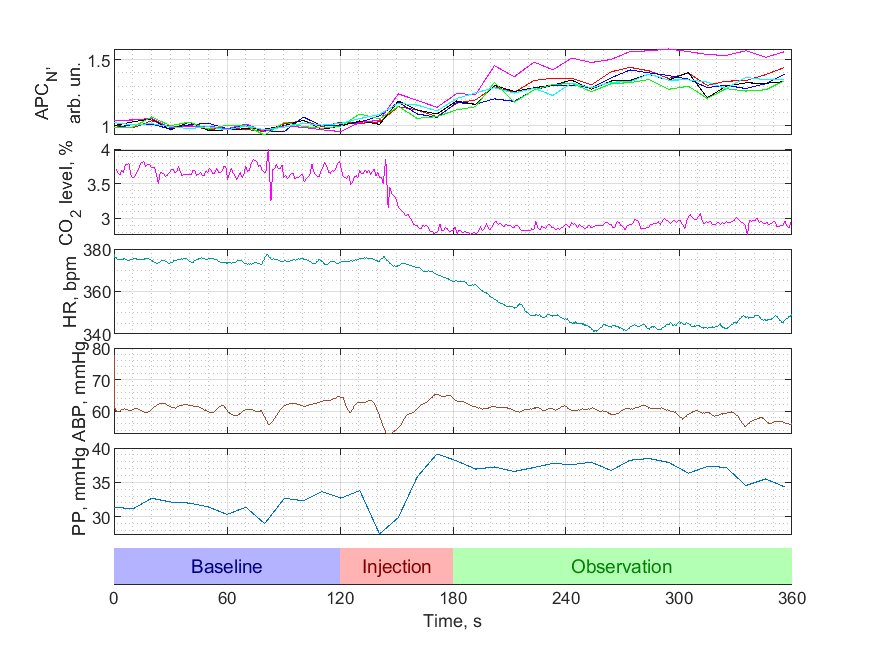


**Figure S11.** Responses of the measured physiological indices on the dorzolamide injection for the Rat #11 (measured at 31/07/2019, weight: 409 g)


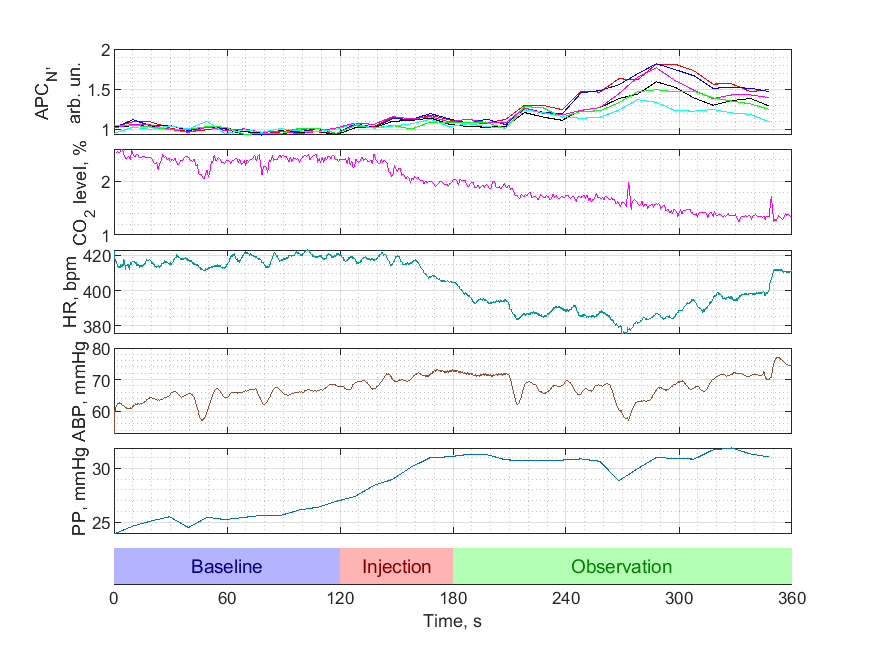


**Figure S12.** Responses of the measured physiological indices on the dorzolamide injection for the Rat #12 (measured at 05/08/2019, weight: 400 g)


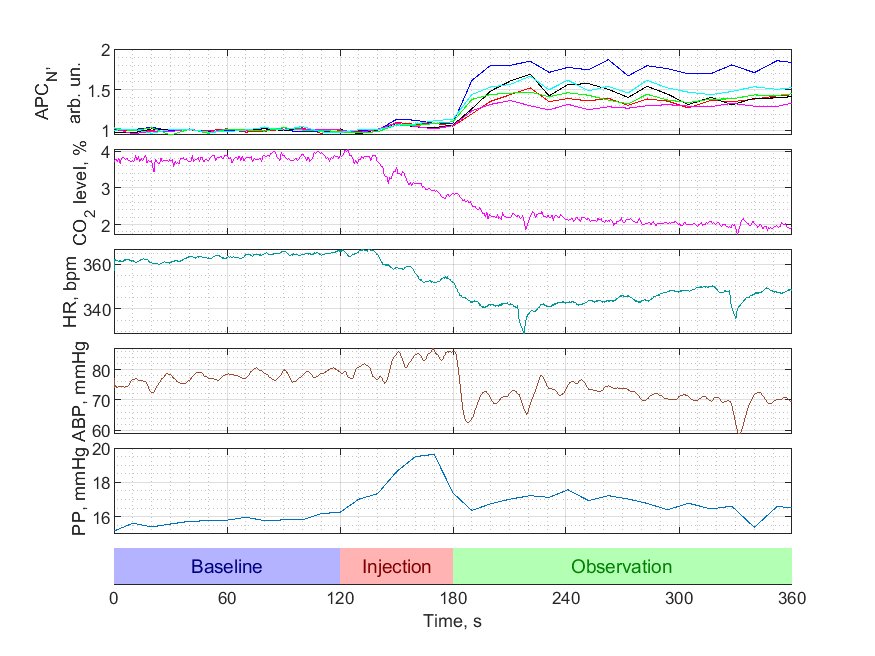


**Figure S13.** Responses of the measured physiological indices on the dorzolamide injection for the Rat #13 (measured at 14/08/2019, weight: 458 g)


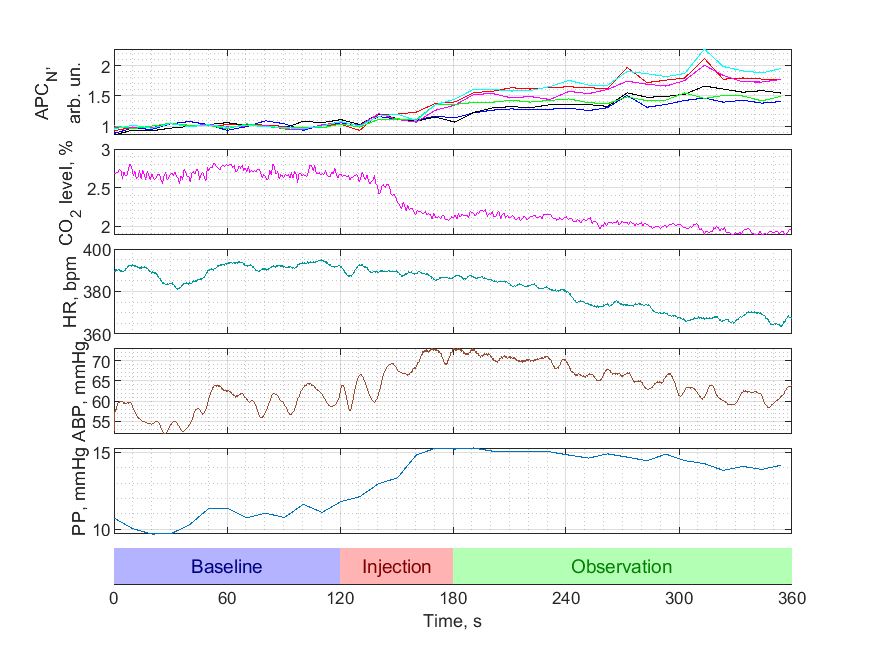


**Figure S14.** Responses of the measured physiological indices on the dorzolamide injection for the Rat #14 (measured at 19/08/2019, weight: 440 g)


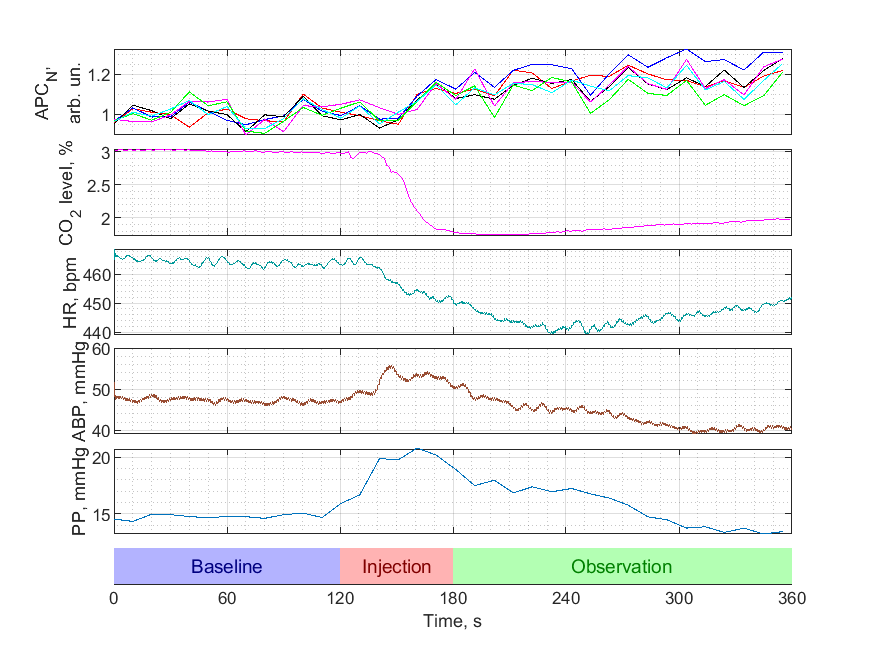


**Figure S15.** Responses of the measured physiological indices on the dorzolamide injection for the Rat #15 (measured at 29/08/2019, weight: 395 g)
